# Letter to the editor regarding “Neuroprogression in post-traumatic stress disorder: a systematic review”

**DOI:** 10.47626/2237-6089-2021-0360

**Published:** 2021-11-30

**Authors:** Joana Bücker

**Affiliations:** 1 Programa de Pós-Graduação em Ciências Médicas Universidade do Vale do Taquari Lajeado RS Brazil Programa de Pós-Graduação em Ciências Médicas, Universidade do Vale do Taquari (UNIVATES), Lajeado, RS, Brazil.

In the systematic review by Antonelli-Salgado, et al.,^1^ published in a previous issue of Trends in Psychiatry and Psychotherapy, the authors assessed the outcomes associated with neuroprogression in post-traumatic stress disorder (PTSD) patients. They concluded that, despite a progressive change in the frontal lobe, neurocognition, and functioning in these patients, further research is needed to characterize PTSD as a neuroprogressive disorder. This study has various strengths, such as being the first systematic review of neuroprogression assessment in PTSD through brain anatomy, neurocognition, functioning, inflammation, oxidative stress, and neurotrophins. However, it would be interesting to extend the original article’s argument using complementary information^2^ that may help clarify the neuroprogression hypothesis in PTSD.

Despite the authors’ statement that progressive brain reduction is associated with cognitive impairment in PTSD patients, specific important aspects of cognitive function such as emotional memory were not examined in their review. Emotional memory is an important cognitive function characterized by enhanced memory for emotional events. It seems to be associated with a dysfunctional hyperactivation of the amygdala, which plays an important key role in the pathophysiology of PTSD,^1^ making patients more susceptible to interpreting neutral life events as traumatic. Therefore, studying the cognitive-emotional processing in individuals with PTSD is essential to better understand the possible progressive condition associated with this disorder.

Another important point to consider is the fact that, over the last few years, a growing body of evidence has shown that emotional memory may be involved in the neuroprogression of psychiatric disorders, especially in bipolar disorder patients;^3^ since bipolar disorder and PTSD are common comorbidities and have similarities in terms of biological alterations,^1^ so it would be important to investigate emotional memory as an outcome associated with neuroprogression in PTSD as well.

Moreover, previous research has shown that emotional memory is altered in PTSD. A recent systematic review revealed an increased recall for negative emotional stimuli in these patients,^4^ suggesting that emotional memory might play an important role in PTSD pathophysiology. The authors found an increased recall for negative stimuli, decreased recall for neutral stimuli, and activation of specific brain areas during emotional tasks in PTSD patients. Other than that, a previous longitudinal study^5^ evaluated emotional memory with a face memory task in PTSD patients and revealed that the amygdala (as a function of emotional memory) might be a marker of symptom severity, and the activity in the subgenual anterior cingulate cortex (as a function of emotional memory) might be associated with recovery. Given this, it is suggested that investigating the influence of emotional memory in maintenance and trajectory of PTSD could be beneficial to understand the neuroprogression hypothesis in this field.
